# Supplementary material for: MetaRibo-Seq measures translation in microbiomes
Source: Nat Commun. 2020 Jun 29;11:3268. doi: 10.1038/s41467-020-17081-z (PMC7324362; doi:10.1038/s41467-020-17081-z)
Supplement: Supplementary file 10 — Supplementary Data 7 [file 41467_2020_17081_MOESM10_ESM.zip › File2/Confidence_High_Taxonomy/35054_out.krona.html]

Javascript must be enabled to view this page.

members
magnitude
magnitudeUnassigned
count
unassigned
taxon
rank

35054\_out

2

2
superkingdom
2

2
32066
phylum

2
class
203490

203491
order
2

2
family
1129771

2
32067
genus


SRS015686\_contig\_number\_8328SRS024637\_contig\_number\_contig-100\_3758.3758.3758
157687
species
2
